# Supplementary material for: Risk factors for repeat self-harm hospitalisation following hospital admissions for suicidal ideation and self-harm among Aboriginal and non-Aboriginal people: a retrospective cohort study using linked administrative data
Source: Soc Psychiatry Psychiatr Epidemiol. 2026 Feb 27;61(9):1727–38. doi: 10.1007/s00127-026-03065-9 (PMC13578029; doi:10.1007/s00127-026-03065-9)
Supplement: Supplementary file 1 [file 127_2026_3065_MOESM1_ESM.docx]

Long-term risk of repeat hospital admission involving self-harm by Aboriginal and non-Aboriginal people: A retrospective cohort study

Social Psychiatry and Psychiatric Epidemiology

Bernard Leckning, Rohan Borschmann, Tanja Hirvonen, Sven R. Silburn, Steven Guthridge, Gary W. Robinson

# Supporting information

S1 Table. Descriptive summary and univariate analysis of repeat hospital admission involving self-harm by Aboriginal people with a hospital admission involving self-harm and suicidal ideation, NT 2001-2013

|  |  | **Any repeat self-harm?** | | | |  |
| --- | --- | --- | --- | --- | --- | --- |
|  |  | **No** | **Yes** | **β** | **p-value** |  |
|  |  | **%** | **%** |  |  |  |
|  |  |  |  |  |  |  |
| **Total (n=2,304)** |  | **75%** | **25%** |  |  |  |
| **Demographic characteristics at index admission** | | | | | | |
| *Sex* |  |  |  |  |  |  |
| Female (n=1,131) |  | 75% | 25% | REF | . |  |
| Male (n=1,173) |  | 76% | 24% | -0.070 | 0.402 |  |
| *Age in years* |  |  |  | 0.001 | 0.746 |  |
| 10-19 (n=425) |  | 77% | 23% |  |  |  |
| 20-34 (n=1,181) |  | 75% | 25% |  |  |  |
| 35-54 (n=663) |  | 75% | 25% |  |  |  |
| 55+ (n=35) |  | 80% | 20% |  |  |  |
| *Residence (hospital district)* |  |  |  |  |  |  |
| Darwin Urban (n=383) |  | 76% | 24% | REF | . |  |
| Darwin Rural (n=242) |  | 83% | 17% | -0.412 | 0.030 |  |
| East Arnhem District (n=318) |  | 76% | 24% | -0.002 | 0.989 |  |
| Katherine District (n=311) |  | 77% | 23% | -0.072 | 0.651 |  |
| Alice Springs Urban (n=427) |  | 70% | 30% | 0.314 | 0.023 |  |
| Alice Springs Rural (n=406) |  | 74% | 26% | 0.134 | 0.349 |  |
| Barkly District (n=217) |  | 74% | 26% | 0.147 | 0.386 |  |
| *Residence (remoteness)* |  |  |  |  |  |  |
| Urban (n=804) |  | 74% | 26% | REF | . |  |
| Remote (n=1,500) |  | 76% | 24% | -0.152 | 0.079 |  |
| *Residence (region)* |  | 0% | 0% |  |  |  |
| Top End (n=1,254) |  | 78% | 22% | REF | . |  |
| Central Aust. (n=1,050) |  | 72% | 28% | 0.300 | 0.000 |  |
| *Residence (region x remoteness)* | | | | | | |
| Top End Urban (n=376) |  | 77% | 23% | REF | . |  |
| Top End Remote (n=878) |  | 78% | 22% | -0.064 | 0.622 |  |
| Cent. Aust. Urban (n=428) |  | 70% | 30% | 0.354 | 0.011 |  |
| Cent. Aust. Remote (n=622) |  | 74% | 26% | 0.185 | 0.165 |  |
| **Clinical characteristics at index admission** | | | | | | |
| *Type of suicidal behavior* |  |  |  |  |  |  |
| Suicidal ideation (n=628) |  | 84% | 16% | REF | . |  |
| Self-harm (n=1,676) |  | 72% | 28% | 0.548 | 0.000 |  |
| Self-poisoning (n=440) |  | 74% | 26% | 0.427 | 0.002 |  |
| Self-cutting (n=718) |  | 67% | 33% | 0.758 | 0.000 |  |
| Hanging (n=379) |  | 79% | 21% | 0.253 | 0.091 |  |
| Other methods (n=139) |  | 74% | 26% | 0.466 | 0.017 |  |
| Any mental health diagnosis | No (n=695) | 76% | 24% | REF | . |  |
|  | Yes (n=1,609) | 75% | 25% | 0.106 | 0.254 |  |
| Severe mental disorders | No (n=2,125) | 75% | 25% | REF | . |  |
|  | Yes (n=179) | 79% | 21% | -0.154 | 0.364 |  |
| Schizophrenia and other psychotic disorders | No (n=2,212) | 75% | 25% |  |  |  |
|  | Yes (n=92) | 80% | 20% |  |  |  |
| Schizoaffective disorders | No (n=2,294) | 75% | 25% |  |  |  |
|  | Yes (n=10) | 70% | 30% |  |  |  |
| Psychotic affective disorders | No (n=2,285) | 75% | 25% |  |  |  |
|  | Yes (n=19) | 79% | 21% |  |  |  |
| Psychotic disorders related to substance use | No (n=2,233) | 75% | 25% |  |  |  |
|  | Yes (n=71) | 83% | 17% |  |  |  |
| Common mental disorders | No (n=1,647) | 74% | 26% | REF | . |  |
|  | Yes (n=657) | 78% | 22% | -0.166 | 0.084 |  |
| Non-psychotic affective disorders | No (n=2,044) | 75% | 25% |  |  |  |
|  | Yes (n=260) | 75% | 25% |  |  |  |
| Neurotic and stress-related disorders | No (n=1,844) | 75% | 25% |  |  |  |
|  | Yes (n=460) | 78% | 22% |  |  |  |
| Personality disorders | No (n=2,265) | 76% | 24% | REF | . |  |
|  | Yes (n=39) | 62% | 38% | 0.581 | 0.027 |  |
| Personality disorders (A) | No (n=2,302) | 75% | 25% |  |  |  |
|  | Yes (n=2) | 0% | 100% |  |  |  |
| Personality disorders (B) | No (n=2,277) | 76% | 24% |  |  |  |
|  | Yes (n=27) | 56% | 44% |  |  |  |
| Personality disorders (C) | No (n=2,298) | 75% | 25% |  |  |  |
|  | Yes (n=6) | 100% | 0% |  |  |  |
| Other personality disorders | No (n=2,298) | 75% | 25% |  |  |  |
|  | Yes (n=6) | 67% | 33% |  |  |  |
| Substance use disorders | No (n=1,176) | 78% | 22% | REF | . |  |
|  | Yes (n=1,128) | 72% | 28% | 0.315 | 0.000 |  |
| Alcohol intoxication and use disorders | No (n=1,280) | 78% | 22% | REF | . |  |
|  | Yes (n=1,024) | 72% | 28% | 0.356 | 0.000 |  |
| Other substance intoxication and use disorders | No (n=2,059) | 75% | 25% | REF | . |  |
|  | Yes (n=245) | 78% | 22% | -0.113 | 0.427 |  |
| **Clinical characteristics from previous admissions** | | | | | | |
| Any mental health diagnosis | No (n=1,839) | 78% | 22% | REF | . |  |
|  | Yes (n=465) | 65% | 35% | 0.606 | 0.000 |  |
| Severe mental disorders | No (n=2,235) | 76% | 24% | REF | . |  |
|  | Yes (n=69) | 71% | 29% | 0.195 | 0.393 |  |
| Common mental disorders | No (n=2,232) | 76% | 24% | REF | . |  |
|  | Yes (n=72) | 68% | 32% | 0.390 | 0.067 |  |
| Personality disorders | No (n=2,295) | 76% | 24% | REF | . |  |
|  | Yes (n=9) | 44% | 56% | 1.189 | 0.008 |  |
| Substance use disorders | No (n=1,897) | 78% | 22% | REF | . |  |
|  | Yes (n=407) | 63% | 37% | 0.677 | 0.000 |  |
| Injury | No (n=1,969) | 77% | 23% | REF | . |  |
|  | Yes (n=335) | 67% | 33% | 0.379 | 0.069 |  |

S2 Table. Descriptive summary and univariate analysis of repeat hospital admission involving self-harm by non-Aboriginal people with a hospital admission involving self-harm and suicidal ideation

|  |  | **Any repeat self-harm?** | | | |
| --- | --- | --- | --- | --- | --- |
|  |  | **No** | **Yes** | **β** | **p-value** |
|  |  | **%** | **%** |  |  |
|  |  |  |  |  |  |
| **Total (n=2,304)** |  | **75%** | **25%** |  |  |
| **Demographic characteristics at index admission** | | | | | |
| *Sex* |  |  |  |  |  |
| Female (n=1,131) |  | 75% | 25% | REF | . |
| Male (n=1,173) |  | 76% | 24% | -0.070 | 0.402 |
| *Age in years* |  |  |  | 0.001 | 0.746 |
| 10-19 (n=425) |  | 77% | 23% |  |  |
| 20-34 (n=1,181) |  | 75% | 25% |  |  |
| 35-54 (n=663) |  | 75% | 25% |  |  |
| 55+ (n=35) |  | 80% | 20% |  |  |
| *Residence (hospital district)* |  |  |  |  |  |
| Darwin Urban (n=383) |  | 76% | 24% | REF | . |
| Darwin Rural (n=242) |  | 83% | 17% | -0.412 | 0.030 |
| East Arnhem District (n=318) |  | 76% | 24% | -0.002 | 0.989 |
| Katherine District (n=311) |  | 77% | 23% | -0.072 | 0.651 |
| Alice Springs Urban (n=427) |  | 70% | 30% | 0.314 | 0.023 |
| Alice Springs Rural (n=406) |  | 74% | 26% | 0.134 | 0.349 |
| Barkly District (n=217) |  | 74% | 26% | 0.147 | 0.386 |
| *Residence (remoteness)* |  |  |  |  |  |
| Urban (n=804) |  | 74% | 26% | REF | . |
| Remote (n=1,500) |  | 76% | 24% | -0.152 | 0.079 |
| *Residence (region)* |  | *0%* | *0%* |  |  |
| Top End (n=1,254) |  | 78% | 22% | REF | . |
| Central Aust. (n=1,050) |  | 72% | 28% | 0.300 | 0.000 |
| *Residence (region x remoteness)* |  |  |  |  |  |
| Top End Urban (n=376) |  | 77% | 23% | REF | . |
| Top End Remote (n=878) |  | 78% | 22% | -0.064 | 0.622 |
| Cent. Aust. Urban (n=428) |  | 70% | 30% | 0.354 | 0.011 |
| Cent. Aust. Remote (n=622) |  | 74% | 26% | 0.185 | 0.165 |
| **Clinical characteristics at index admission** | | | | | |
| *Type of suicidal behavior* |  |  |  |  |  |
| Suicidal ideation (n=628) |  | 84% | 16% | REF | . |
| Self-harm (n=1,676) |  | 72% | 28% | 0.548 | 0.000 |
| Self-poisoning (n=440) |  | 74% | 26% | 0.427 | 0.002 |
| Self-cutting (n=718) |  | 67% | 33% | 0.758 | 0.000 |
| Hanging (n=379) |  | 79% | 21% | 0.253 | 0.091 |
| Other methods (n=139) |  | 74% | 26% | 0.466 | 0.017 |
| Any mental health diagnosis | No (n=695) | 76% | 24% | REF | . |
|  | Yes (n=1,609) | 75% | 25% | 0.106 | 0.254 |
| Severe mental disorders | No (n=2,125) | 75% | 25% | REF | . |
|  | Yes (n=179) | 79% | 21% | -0.154 | 0.364 |
| Schizophrenia and other psychotic disorders | No (n=2,212) | 75% | 25% |  |  |
|  | Yes (n=92) | 80% | 20% |  |  |
| Schizoaffective disorders | No (n=2,294) | 75% | 25% |  |  |
|  | Yes (n=10) | 70% | 30% |  |  |
| Psychotic affective disorders | No (n=2,285) | 75% | 25% |  |  |
|  | Yes (n=19) | 79% | 21% |  |  |
| Psychotic disorders related to substance use | No (n=2,233) | 75% | 25% |  |  |
|  | Yes (n=71) | 83% | 17% |  |  |
| Common mental disorders | No (n=1,647) | 74% | 26% | REF | . |
|  | Yes (n=657) | 78% | 22% | -0.166 | 0.084 |
| Non-psychotic affective disorders | No (n=2,044) | 75% | 25% |  |  |
|  | Yes (n=260) | 75% | 25% |  |  |
| Neurotic and stress-related disorders | No (n=1,844) | 75% | 25% |  |  |
|  | Yes (n=460) | 78% | 22% |  |  |
| Personality disorders | No (n=2,265) | 76% | 24% | REF | . |
|  | Yes (n=39) | 62% | 38% | 0.581 | 0.027 |
| Personality disorders (A) | No (n=2,302) | 75% | 25% |  |  |
|  | Yes (n=2) | 0% | 100% |  |  |
| Personality disorders (B) | No (n=2,277) | 76% | 24% |  |  |
|  | Yes (n=27) | 56% | 44% |  |  |
| Personality disorders (C) | No (n=2,298) | 75% | 25% |  |  |
|  | Yes (n=6) | 100% | 0% |  |  |
| Other personality disorders | No (n=2,298) | 75% | 25% |  |  |
|  | Yes (n=6) | 67% | 33% |  |  |
| Substance use disorders | No (n=1,176) | 78% | 22% | REF | . |
|  | Yes (n=1,128) | 72% | 28% | 0.315 | 0.000 |
| Alcohol intoxication and use disorders | No (n=1,280) | 78% | 22% | REF | . |
|  | Yes (n=1,024) | 72% | 28% | 0.356 | 0.000 |
| Other substance intoxication and use disorders | No (n=2,059) | 75% | 25% | REF | . |
|  | Yes (n=245) | 78% | 22% | -0.113 | 0.427 |
| **Clinical characteristics from previous admissions** | | | | | |
| Mental health & behavioral disorder | No (n=1,839) | 78% | 22% | REF | . |
|  | Yes (n=465) | 65% | 35% | 0.606 | 0.000 |
| Severe mental disorders | No (n=2,235) | 76% | 24% | REF | . |
|  | Yes (n=69) | 71% | 29% | 0.195 | 0.393 |
| Common mental disorders | No (n=2,232) | 76% | 24% | REF | . |
|  | Yes (n=72) | 68% | 32% | 0.390 | 0.067 |
| Personality disorders | No (n=2,295) | 76% | 24% | REF | . |
|  | Yes (n=9) | 44% | 56% | 1.189 | 0.008 |
| Substance use disorders | No (n=1,897) | 78% | 22% | REF | . |
|  | Yes (n=407) | 63% | 37% | 0.677 | 0.000 |
| Injury | No (n=1,969) | 77% | 23% | REF | . |
|  | Yes (n=335) | 67% | 33% | 0.379 | 0.069 |

S3 Table. Statistical tests of zero slope of Schoenfeld residuals over time for model involving Aboriginal individuals

|  |  | **Rho** | **Chi^2^** | **DF** | **P value** |
| --- | --- | --- | --- | --- | --- |
| **Demographic characteristics at index admission** |  |  |  |  |  |
| *Sex* |  |  |  |  |  |
| Female |  | . | . | 1 | . |
| Male |  | -0.02698 | 0.42 | 1 | 0.5194 |
| Age in years (median centred) |  | -0.03091 | 0.47 | 1 | 0.4948 |
| *Residence* |  |  |  |  |  |
| Top End |  | . | . | 1 | . |
| Central Australia |  | -0.00481 | 0.01 | 1 | 0.9069 |
| **Clinical characteristics at index admission** |  |  |  |  |  |
| *Type of suicidal behaviour* |  |  |  |  |  |
| Suicidal ideation |  | . | . | 1 | . |
| Self-harm |  |  |  | 1 |  |
| Self-poisoning |  | -0.09648 | 5.22 | 1 | 0.0224 |
| Self-cutting |  | -0.03148 | 0.58 | 1 | 0.4450 |
| Hanging |  | -0.01520 | 0.13 | 1 | 0.7190 |
| Other self-harm |  | -0.04652 | 1.22 | 1 | 0.2693 |
| Alcohol use disorders | No | . | . | 1 | . |
|  | Yes | -0.03899 | 0.89 | 1 | 0.3443 |
| **Clinical characteristics from previous admissions** |  |  |  |  |  |
| Alcohol use disorders | No | . | . | 1 | . |
|  | Yes | -0.02324 | 0.30 | 1 | 0.5822 |
| **Global test** |  |  | 8.66 |  | 0.4695 |

S4 Table. Statistical tests of zero slope of Schoenfeld residuals over time for model involving non-Aboriginal individuals

|  |  | **Rho** | **Chi^2^** | **DF** | **P value** |
| --- | --- | --- | --- | --- | --- |
| **Demographic characteristics at index admission** |  |  |  |  |  |
| *Sex* |  |  |  |  |  |
| Female |  | . | . | 1 | . |
| Male |  | -0.15111 | 8,75 | 1 | 0.0031 |
| Age in years (median centred) |  | 0.08119 | 2.31 | 1 | 0.1282 |
| *Residence* |  |  |  |  |  |
| Top End |  | . | . | 1 | . |
| Central Australia |  | -0.04352 | 0.71 | 1 | 0.3992 |
| **Clinical characteristics at index admission** |  |  |  |  |  |
| *Type of suicidal behaviour* |  |  |  |  |  |
| Suicidal ideation |  | . | . | 1 | . |
| Self-harm |  |  |  |  |  |
| Self-poisoning |  | 0.00576 | 0.01 | 1 | 0.9101 |
| Self-cutting |  | -0.03279 | 0.42 | 1 | 0.5188 |
| Hanging |  | -0.00260 | 0.00 | 1 | 0.9601 |
| Other self-harm |  | -0.03340 | 0.42 | 1 | 0.5184 |
| Severe mental disorders | No | . | . | 1 | . |
|  | Yes | -0.03262 | 0.40 | 1 | 0.5263 |
| Personality disorders | No | . | . | 1 | . |
|  | Yes | 0.01278 | 0.06 | 1 | 0.8061 |
| Alcohol use disorders | No | . | . | 1 | . |
|  | Yes | 0.14070 | 7.47 | 1 | 0.0063 |
| **Clinical characteristics from previous admissions** |  |  |  |  |  |
| Common mental disorders | No | . | . | 1 | . |
|  | Yes | -0.08893 | 3.21 | 1 | 0.0731 |
| Substance use disorders | No | . | . | 1 | . |
|  | Yes | -0.00383 | 0.01 | 1 | 0.9393 |
| **Global test** |  |  | 22.67 | 12 | 0.0307 |

Figure S1. Log-log plot by sex from the model involving non-Aboriginal individuals
